# Cyclopædiæ and Dictionaries of Medicine and Surgery

**Published:** 1837-07-01

**Authors:** 


### CYCLOPÆDIÆ AND DICTIONARIES OF MEDICINE AND SURGERY.

I. A Dictionary of Practical Medicine, &c. By *James Copland*, M.D. &c. Part IV. 8vo. Pp. 304, London, 1837. Price Nine Shillings.

II. The American Cyclopædia of Practical Medicine and Surgery, &c. Edited by *Isaac Hays*, M.D., &c. Part X. Philadelphia, September, 1836.

III. The Cyclopædia of Practical Surgery, comprising a Series of Original Dissertations on Operative Medicine, by an Association of Physicians and Surgeons. Edited by *W. B. Costello*, M.D., &c. Part I. London, 1837.

At first sight, it appears evident that a Cyclopædia written by numerous individuals, is preferable to a Dictionary composed by one. It seems indisputable that, when each contributor is chosen for his knowledge of the subject on which he is to write, the result will be a collection of the most valuable essays, and an amount of information will be obtained in this manner, which no one person, however great his skill or learning, could possibly convey.

But the advantages so obviously attending a cyclopædia of general science, are not found to flow in the same degree from that form of publication on the practice of medicine. The most experienced and most eminent in the profession are too much and too profitably occupied, to enable them to devote their time to the composition of articles, for which no commensurate remuneration can be given. Unfortunately too, there are so many subjects on which a wide difference of sentiment exists, that when many surgeons or physicians write, almost as many contradictory opinions will be uttered; and the reader of a cyclopædia who finds one doctrine laid down in one part, may stumble on the opposite dogma in another. An able physician in this metropolis has said with much naiveté, perhaps with some truth, that the Cyclopædia of Practical Medicine, is a large book in which every man has ridden his own hobby.

It is no doubt owing to these, and, possibly, to other circumstances, that, practically, medical cyclopædias are not found to excel, in the degree we might anticipate, those dictionaries which have been the work of but one individual. If the latter want variety, they are characterised by unity of design and of construction, more useful perhaps to the reader and the student. The Dictionary of Dr. Copland has appeared since the Cyclopædia of Medicine was published. It had, therefore, to sustain a comparison with its rival, and to struggle with the disadvantage of being late in the market. Yet its success has been remarkable, and it may be doubted whether it will not ultimately beat the Cyclopædia.

Of course, we have no intention of presenting an analysis of any of the works which are lying before us. Their elaborate and elementary character preclude the possibility of that. We shall merely give a bird's-eye view of their contents, and select some articles for a special notice.

1. The present, the fourth part of the Dictionary of Practical Medicine, commences with the conclusion of the subject of Remittent Fever, and terminates with the treatment of Hypertrophy of the Heart. It embraces the consideration of the following important maladies—of Hectic and Continued Fevers—of Fungoid Diseases—of Gangrene—Gout—Hæmorrhage—and of some of the Diseases of the Heart.

We must admit that we have not been enabled to read 304 pages, printed closely in double columns, nor can we pretend to select a thousandth part of what is really valuable. But the wide circulation of the Dictionary both excuses our silence, and forbids the attempt to extract much from its pages; while the ability and care with which its articles have been composed, display an uniformity of excellence which renders selection as unnecessary as it is difficult.

We may take, almost at random, the subject of deafness, and exhibit the manner in which it is treated by our able friend. We do so, because it offers a striking proof of his diligence and judgment, and because our readers may be nothing the worse for seeing, in a connected series, the many causes and the appropriate treatment of that troublesome disorder.

Dr. Copland arranges the causes of deafness under four heads:—1, deafness from affections of the external ear—2, deafness from disease of the Eustachian tube and cavity of the tympanum—3, deafness from affections of the auditory nerves—4, deafness and dumbness.

*Deafness from Affections of the External Ear.*—A. Diseases of the auricle, occasionally swelling, as erysipelatous inflammation, or scirrhus may occasion deafness.

B. It is more likely to result from diseases of the auditory passage and membrane of the tympanum. All the affections of these parts are inflammatory, or the consequences of inflammation, and are thus arranged.

*Erythematic Inflammation of the Auditory Passage.*—This generally causes accumulations of brownish hard wax, obstructing more or less the functions of the organ. It sometimes occurs in persons of a cachectic habit of body, or in conjunction with chronic affections of the skin, and in connexion with disorder of the digestive and excreting organs. It is often excited by substances that have passed into the ear, or by neglect of cleanliness, which, however, is not so frequent a cause as is generally supposed; the accumulation of hardened or morbid wax, with increased sensibility, pain, or soreness in the meatus, being the chief indications of the affection. In its slighter states, itching or formication in the passage is only felt.

The *treatment* of this affection consists in attention to the secretions, and in syringing the meatus with tepid water.

*Inflammation of the Follicles of the Auditory Passage.*—"From this inflammation, and from that of the *membrana tympani*, various excrescences or morbid growths in the meatus ultimately proceed. On inspection redness and partial swelling of the walls of the passage are first observed; and, if the affection continues long, or becomes chronic, excrescences, or polypi, of a soft, spongy, or vesicular appearance, are gradually formed. These are red, sensitive, roundish, pedunculated, and readily bleed when irritated. In some cases, they have a broad hard base, are insensible, and not disposed to bleed. These obstruct more or less, the meatus, and impede the functions of the organ. Hardened mucus and wax may also accumulate in the passage, as a consequence of the chronic states of this affection, and of the obstruction caused by these excrescences."

The *treatment* consists in attention to the health—syringing the meatus—extirpation of the fungous growth; and, should the membrana tympani be thickened, or ulcerated, or covered with inspissated secretions, in employing the means for otorrhœa, viz. blisters, setons, or issues as circumstances may require.

*Phlegmonous Inflammation of the Passage.*—This may be readily distinguished, and its treatment must be obvious.

*Inflammation of the Periosteum of the Passage.*—" This is most common in children of a scrofulous diathesis, and generally occasions caries of the bony structure, which is readily detected with the probe. If exfoliation of the diseased bone occur, and the ulcerated part begins to heal, narrowing or obliteration of the meatus may take place. In these cases, the deafness often depends as much upon congestion of the adjoining parts, as upon swelling and disease of the passage.—Dr. Kramer advises, in the *treatment*, that, when the parts show a tendency to close, they should be opened up by art, and maintained open by touching them with lunar caustic, throughout their extent. Hearing, however, usually continues very dull, owing to the natural form of the meatus having been lost, and to the membrane of the drum having become thickened."

*The Diseases of the Membrane of the Tympanum* are, in some instances, a dubious, and in others an indubitable cause of deafness. It is not, for example, certain that relaxation of that membrane, nor undue tension of it, nor rupture of it, nor rupture of the tendon of the tensor tympani, frequently give rise to deafness. When the membrane is ruptured indeed, it is usually rather from an ulcerative process.

*Inflammation*, then, of the membrane of the tympanum, however it has been induced, may end in ulceration or in other lesions.

" Acute inflammation of this part is not so common as the sub-acute and chronic states; and either, when neglected, gives rise to opacity, thickening, perforation, purulent discharge, fungous or polypous excrescences, &c.; but the chronic states most frequently induce these lesions. In acute inflammation, the membrane is seen, on careful examination, more or less red, rough, swollen, and opaque. It often seems as if covered with small projecting glands or follicles. Sometimes bundles of vessels are seen in it, and the point of insertion of the handle of the malleus cannot be distinguished, Dr. Kramer states, that inflammation of this part are distinguished from internal inflammations of the ear, not only by the greater mildness of the former, but especially by the changes of the membrane presented by them from the commencement; whereas, in the latter, such changes cannot be detected early in the disease, however violent the symptoms and attendant fever may be; and occur only in the further course of the malady, when the membrane is about to burst from the pressure of accumulated matter, or has become involved in the inflammatory process. The different grades of this disease have been imputed to *nervous otalgia*, or confounded with it. Dr. Kramer, however, denies the existence of such an affection. In this he is evidently mistaken; although it must be admitted, that both this, and other inflammatory diseases of the ear, are often improperly viewed as nervous merely. The hardened secretion in the meatus, to which the more chronic states of inflammation of the membrane have been imputed, is more commonly the result of inflammatory action, than its cause. The disease, in both its primary and its consecutive states, generally impairs hearing more or less." 158.

This inflammation is to be met by such appropriate treatment as we need not stop to consider. In addition, various injections have been recommended—a solution of the acetate of lead—of sulphate of zinc—of nitrate of silver, and so forth. The surgeon should be cautious. We have seen some mischief, and heard of more, from employing injections of too stimulating a description. A few drops of the pyroligneous acid to an ounce of water, as well as the solution of the acetate of lead will effectually remove the offensive odour of the discharge.

*Perforation of the membrane*, when to any extent, must materially impair the hearing. But there are certain states of the ear in which artificial perforation is of service. Itard, observes Dr. Copland, contends, that the operation is admissible only when there is invincible obstruction in the tube; Saissy advises it only in thickening and hardening of the membrane; and Deleau recommends it also in this case, as well as in obstruction or obliteration of the Eustachian tube, and in obstruction of the cavity of the tympanum. Dr. Kramer has recourse to the operation only when the *membrana tympani* is much thickened, quite insensible to the probe, hard as cartilage, and if the hearing is very impaired; but, even in this case, it should be performed only when both ears are affected with considerable deafness, and when the ear to be operated upon does not suffer from any other disease, by which the result might be rendered abortive. Slight obstruction of the Eustachian tube is undoubtedly to be treated, not by perforating the membrane of the tympanum, but by the introduction of instruments into the tube itself.

2. *Deafness from Disease of the Eustachian Tube and Cavity of the Tympanum.* A. The tube may be obstructed, 1st, by the pressure of tumors in its vicinity;—2d, by inflammation causing tumefaction of the mucous membrane, effusion, &c.;—and, 3d, by the more remote consequences of inflammation, namely, constriction or obliteration of a portion, or of the whole, of the canal. To ascertain any of these conditions accurately, it is necessary to explore the tube. This may be done by the introduction of bougies or catheters into it, and by injections of tepid water or of air into the tympanum. Dr. Kramer describes a mode of introducing compressed air into the tympanum, and points out its application as a means of diagnosis. We subjoin the extract taken by our author from Dr. Kramer's observations.

" 'After the catheter has been introduced into the tube, and fixed by means of a frontlet, the patient is placed close to a table, on which he leans his elbow, holding with the hand of that side the pipe of the air-press filled with compressed air. The operator then introduces the metal beak of the pipe into the funnel-shaped dilatation of the catheter, applies his ear close to that which is being examined, opens the cock of the machine and listens to the sound, caused by the air rushing into the cavity of the drum. When the tube and cavity are free, the air strikes with an audible shock against the membrane of the tympanum. When the shock is over, or is slight, a blowing or rustling in the ear of the patient is heard, caused by the streaming of the air.' All variations from this sound are morbid, and furnish more or less distinct indications of diseased changes in the organ. If the air-douche does not penetrate to the *membrana tympani*, Dr. KRAMER advises catgut bougies to be used for opening the passage in the tube." 158.

It must be owned that this is rather nice diagnosis, and that it beats auscultation hollow. What an opportunity it would offer to a Quack aurist, for drumming his sagacity and penetration in his patient's ears. The only objection to it as an engine of quackery is, that probably few Charlatans *could* introduce an instrument into the Eustachian tube.

*Inflammation of the Mucous Membrane of the Eustachian Tube* may be catarrhal, the effect of causes usually occasioning catarrhal affections of the throat. The mucus accumulating produces deafness. Of the treatment we need say nothing, save that injections of warm water or of air have been advised.

*Deafness from Inflammation of the Mucous Membrane of the Tube* may proceed from disease of the throat, or of the proper membrane of the drum; and be complicated with either, or with both these diseases. In the case of its connection with lesion in the cavity of the tympanum, it is either associated with, or has followed, acute *otitis* or *otorrhœa.* But when the inflammation is confined to the gutteral part of the canal, deafness is neither great, nor attended by pain in the interior of the ear. The patient hears well at times, but only momentarily. He hears his own voice even worse than that of others: and occasionally has a crackling, gurgling, or detonating sensation in the throat leading to the ear. The diagnosis is still more to be depended upon, if pain or inflammation exists in the throat or fauces, and if the former be increased on gaping or mastication. The chronic states of the disease of the tube are generally connected with syphilis, or with the scrophulous diathesis."

The treatment will depend, in a great measure, on the nature of the affection of the throat. When there is acute inflammation, of course antiphlogistic measures are requisite.

*Diminution, Stricture,* or even *complete Obliteration* of the tube may result from the thickening or ulceration occasioned by the inflammation. This may be the consequence of the cicatrization of an ulcer situated near the orifice of the tube. The diagnosis is important, and is chiefly to be gathered from an enquiry into the history of the case. Exploration of the tube is also an item in the method of investigation. If the obliteration of the canal is complete, the cavity of the drum is always involved in the disease. Perforation of the membrana tympani is said, on this account, to be useless, and these cases are incurable.

*Tumors pressing on the Eustachian Tube* will give rise to deafness. Enlarged tonsils, enlarged parotids, or polypous growths may do so. The diagnosis made, appropriate treatment must be had recourse to.

*Inflammation of the Cavity of the Tympanum.*—The inflammation may affect only the mucous membrane lining this cavity; or it may extend to the submucous cellular tissue, and even to the periosteum. It is generally either acute or chronic; and in either case is a severe, and often dangerous disease. But its consideration belongs rather to otitis than to deafness, and we pass it by.

*Deafness from Extravasation of Blood in the Cavity of the Drum,* occasionally follows violent attacks of sneezing, or constriction of the neck. Inflammation may ensue. Sir A. Cooper has advised puncture of the membrana tympani. Probably the blood would drain off by the Eustachian tube, at least in part.

3. *Deafness from Affections of the Auditory Nerves.* There are no positive means of determining that deafness depends on affections of the nerves. It can only be inferred to do so, when no appreciable alteration of the auditory apparatus exists.

Dr. Kramer divides nervous deafness into two forms—" the one attended by excitement or erethism—the other by torpor. Noise in the ears is always present in the former, but never in the latter. This symptom is often, however, attendant on very different diseases of the ear, but in a very indeterminate and inconstant manner. To determine, therefore, whether deafness with noises in the ear proceeds from disease in the organ, or from nervous affection merely, minute investigation and the means of diagnosis already mentioned must be had recourse to. But these are also requisite in the torpid form of nervous deafness."

It may reasonably be doubted whether this distinction is pathologically just, and whether the diagnosis will commonly be accurate. M. Itard's views on the varieties of nervous deafness are as follows:—1. Deafness may proceed from compression of the acoustic nerve; 2, or from palsy of the nerve, the result either of a severe shock or commotion, of convulsions, of apoplexy, of fever, of sympathy with some other organ, or of organic changes in the nerve itself; 3, or deafness may result from plethora.

A. *Deafness dependent on Compression of the Acoustic Nerve.*—There are, of course, many possible causes of compression of the nerve. DUVERNEY and SANDIFORT found these nerves pressed upon by tumors; and SEVERINUS observed them surrounded by serum and effused blood. ITARD found, in a man who had lost the hearing in the left ear, small tumors lying on the corresponding side of the cerebellum, and nearly two ounces of a thick fluid in the ventricle of the same side. In cases adduced by LIEUTAUD, in several detailed by LALLEMAND, and in some seen by myself, an abscess had formed in the part of the brain adjoining the ear, and by pressure or consequent disorganization, had destroyed the functions of the auditory nerve.

The symptoms are such as indicate cerebral affection; severe and nearly constant headach, vertigo, noise in the ears, impaired sight, and weakness of the mental faculties, especially the memory. The diagnosis is too often dubious. The progress of the case is very slow.

B. *Deafness from Palsy of the Acoustic Nerves.*—The first species of palsy is that resulting from a severe shock to the nerves—as a clap of thunder, the explosion of artillery, and so on. When deafness has been occasioned by loud noises, hearing often returns spontaneously in a few days or weeks. If it continues for some months, it is rarely removed by treatment.

The second species is palsy from convulsions. This is most frequently observed in children under four or five years of age. Many of the cases of deaf-dumbness originate in the convulsions occurring during the first dentition. But the deafness may not be the result of the convulsions; both the one and the other more probably being produced by some lesion at the origin of the acoustic nerves, or by effusion into the fourth ventricle, or by some change at the base of the brain, or about the medulla oblongata. When the loss of hearing is complicated with palsy of one side, or of one limb, the nature of the affection may be inferred; but when this is not the case, and when hearing in both ears is lost, the exact nature or seat of lesion can seldom be determined or even surmised. M. Itard considers deafness occurring in this manner as quite incurable.

Deafness from apoplexy is frequent, and may affect both ears. When on a hemiplegiac side, it is incurable. When the patient is young, and when no palsy succeeds the apoplexy, the deafness may possibly be benefited by nature or by art. The efforts of the latter may be briefly summed up—attention to the general condition of the patient, the application of blisters or of moxas behind the ears, and the vapour of æther or of camphor.

*Deafness is sometimes symptomatic of, or associated with, disorders of the digestive organs.*—In these cases, the affection is generally slight; but it is sometimes very considerable and difficult of removal. Impaired and disordered digestion, deranged biliary secretion and excretion, a foul or loaded tongue, tumid abdomen, a morbid state of the evacuations, and an unhealthy aspect of the countenance and of the general surface, generally characterise this form of deafness. Appropriate treatment of the dyspeptic symptoms is required, and blisters or moxas behind the ears should be combined with it. The disorder, says Dr. Copland, of the digestive organs associated with deafness is sometimes also connected with *difficult dentition,* as justly remarked by Nuck, Hesse, and Itard; and occasionally the impaired digestive, assimilating and excreting functions, of which deafness is symptomatic, gives also rise to the production of *intestinal worms.* In these circumstances, the indications of cure are manifest.

*Idiopathic Paralysis of the Acoustic Nerves* has been defined by Itard to be a want of excitability in them, a loss of their sensibility independently of any of the circumstances already mentioned. M. Itard believes, however, that it may be congenital, or supervene at any period of life; but that it most frequently occurs after forty. It is often accompanied with headach, noise in the ears, and mental inaction. Numbness, or want of sensation in the external ear, is sometimes present. M. Itard has seen the organic sensibility of this part entirely lost in two instances. In old persons, this symptom is often observed in slighter degrees, and is attended by dryness of the meatus. This variety of deafness is generally ameliorated by warm or mild weather, and by loud noises; but, as soon as these cease, the affection returns to its former state. It is *caused,* as well as aggravated, by mental exertion and fatigue; by masturbation, venereal excesses, and other depressants; by exposure to cold, currents of air, and humidity; and by the depressing passions. Its accession is imperceptible, and its progress very slow. Sometimes it continues long stationary; but it is little influenced by treatment. If the patient is not far advanced in life, perhaps counter-irritants, the introduction of stimulating vapors into the Eustachian tube, tonics, or electricity may be productive of some service.

*Deafness may depend on Organic Changes in the Acoustic Nerves.*—Sylvius found them atrophied—Akerman indurated—and Morgagni says that in one case they were wanting.

c. *Deafness from Plethora.*—Congestion of the vessels of the head or of the ear is not unfrequently productive of deafness. The patient complains of headache, vertigo, throbbing noises in the ear or head, and so forth, and the general or local plethora is attended or has been preceded by those circumstances which usually precede or attend the plethoric state. The treatment must be in a great measure regulated by the phenomena of the individual case, and we need not enter on the consideration of its details.

4. *Deafness and Dumbness.*—These usually result from acute or chronic *otitis* in early infancy, occasioning organic changes, particularly in the labyrinth or nerves. When the deafness is congenital, such lesions, or imperfect development may be inferred to have taken place in the fœtus. Deafness and dumbness are never remedied, if the former is congenital. If it has arisen from disease of the ear in infancy, our object must be to discover its cause, and its remediability must depend on the nature of the latter.

The concluding section of this instructive article on deafness, is devoted to the examination of the *remedies recommended for impaired or lost hearing.*

Dr. Copland informs us that his aim, an excellent one, is to rescue the affections of the ear from the quacks who have monopolised them to the exclusion of the regular practitioner. There can be no question that the mere aurists are not calculated, either by their candour or their knowledge, to advance even their special art; and the example of what has been effected for diseases of the eyes, since well-educated surgeons made them a part of their study and practice, may encourage us in a similar attempt on the diseases of the ear. Until the profession takes these to itself, we shall have nothing but a repetition of the same pretension, the same empiricism, the same ill-success, and the same ignorance, which have long distinguished the practice of what is termed "acoustic surgery," because, we suppose, it is surgery of no other sort.

It is absurd to suppose that persons who confine themselves to the study of the diseases of one or two organs, can ever be really well acquainted with them. One organ is so linked with another in the economy, that disease will continually extend from it to other parts, or from other parts to it. To distinguish the case a knowledge of pathology is requisite, and how can a mere oculist or aurist acquire a knowledge of pathology? But we, who are writing for medical men, need not argue a question which their common sense must instantly determine. We have only to rouse them not to recognize the truth of what we urge, but to act upon it. We do hope that the diseases of the ear will receive more general attention than they have done, and it is because we hope so, that we have dwelt upon them now. But to return to the consideration of the remedies that have been proposed for deafness.

A. *Constitutional Means.*—Vascular depletions, when congestion or inflammatory action obtains—purgatives—emetics, useless in nervous deafness, injurious when there is cerebral congestion—tonics and stimulants, when indicated—alteratives and deobstruents—even salivation when there is syphilis—squills when the Eustachian tube is obstructed by mucus—dulcamara, or sulphur, when there are cutaneous eruptions—are each and all remedies which have been used, and may be used again, according to the circumstances of the case.

B. *Local Remedies.*—Electricity, galvanism, and mineral magnetism are said by quacks, and thought by fools, to have done wonders, but are not believed by those who know pathology, to be much better than convenient engines of imposture.

C. Moxas, issues, setons, blisters, and the ointment of tartar emetic, if appropriately used, are valuable local remedies.

D. Masticatories were in vogue, but are not at present thought of. Gargles are sanctioned by reason and experience. Lesions of the Eustachian tube, so frequently originate in affections of the throat and posterior nares, that it is of consequence to attend to the latter. The gargles should be adapted to the condition of the throat, a point on which it is unnecessary to dilate.

E. Drops or injections. If irritating or acrid these must be deemed dangerous. Dr. Turnbull recommends ointments with either *veratria, delphinea,* or *aconitine,* to be rubbed around the ear daily; or four or five drops of a spirituous solution of either of these (gr. ij—iv. to ℥ss. of spirit), to be dropped into the ear. Douches of vapor or of warm water have been employed, and may, in some circumstances, be of service.

F. "*Injections into the Eustachian tube* were first recommended by Guizot; but Cleland, in 1731, first proposed them in a practicable mode, namely, by the nose; and Wathen long afterwards proved that a favourable result might be obtained from the practice. The injection of fluids into the tube was advised by Busson and others, to be performed by filling the mouth with the fluid; and, having firmly closed the lips and nose, by forcing it into the tube.—*Air* has also been directed to be forced into the tube, by Cleland and Sims, in the same way, in order to remove obstructions of it; and the smoke of tobacco has been similarly used, with the intention both of removing obstruction, and of exciting the organ, in nervous deafness, but with very equivocal results: I know one instance in which it proved decidedly injurious. Injections of medicated fluids, of vapour, and of air, into the Eustachian tube, by means of a suitable apparatus, have been severally resorted to by Itard, Deleau, and Kramer.—Besides injecting air as a means of diagnosis, Dr. Kramer throws into the tube, through a catheter introduced into it, the vapour of *acetous æther,* generated in a proper apparatus, at a summer temperature; but confines the practice to cases of nervous deafness characterised by torpor, or those unattended by noises in the ear. He also aids the local means by remedies intended to improve the constitution, and the digestive and other functions." 164.

G. Of Russian vapour-baths, warm, fumigating, or sulphur-baths, we need say no more than that their names will suggest the cases in which they may be resorted to as auxiliaries.

This concludes the subject of deafness. We have gone through it in detail, for the same reason which has prompted Dr. Copland to introduce the article into his Cyclopædia—a wish to diffuse accurate notions on the subject among the mass of the profession. It is one which richly deserves their attention, and which, we would fain hope, will as richly repay it. However that may be, it is high time that, in this country, men of science expend some portion of their leisure and their study on the varied affections of the ear.

We now quit the Dictionary of Dr. Copland, an excellent work which every medical man should possess, but we only quit it for a season. We shall constantly have occasion to turn to its pages, and to avail ourselves of the vast collection of facts and of opinions which the industry, learning, and talent of its author have accumulated.

II. The tenth part of the American Cyclopædia, a work much more comprehensive than any of the medical Cyclopædiæ in this country, commences with the article ascites, and terminates with azygos. As it embraces the whole range of the medical sciences, and includes the materia medica, anatomy, physiology, &c. with surgery and physic, we may conceive without difficulty the extent, and form some idea of the probable value of the work.

Perhaps one of the most elaborate papers in the part before us, is that by Dr. Dunglison upon Asphyxia. The article Axilla is exceedingly well executed, and creditable to the anatomical and surgical knowledge of its author Dr. Geddings. The Atmosphere is ably investigated by Dr. Patterson and Dr. Dunglison, and altogether this part is a good sample of a good work. We hope our American brethren will encourage a publication, which is calculated to diffuse among them an acquaintance with medicine in its numerous and varied bearings.*

III. A new candidate for fame and fortune has started up in the Cyclopædia of Practical Surgery, edited by Mr. Costello. It has been doubted by some whether such a cyclopædia is required, whilst the Surgical Dictionary of Mr. Samuel Cooper enjoys the advantage of the superintendence and occasional revision of its author. For our own parts, we think there *is* room for another and more elaborate work. Excellent as are many parts of Mr. Cooper's Dictionary, there are many others which do not offer a sufficient or perhaps an accurate account of what is known by the best informed surgeons of the day, and a Cyclopædia which would really represent existing knowledge, would be, in our opinion, a desideratum. Whether the Cyclopædia before us will answer the expectations which have been excited, and supply the want we have alluded to, will be apparent after a few numbers have been published. We are sure that the editor has exerted himself greatly to secure the co-operation of the ablest hands, and if industry can ensure success, his labours ought to be successful.

The aim of the editor has been to secure a practical rather than a learned series of articles—an aim agreeable to the tastes of professional men in this country, whose habits of utilitarianism lead them to prefer what is of immediate and palpable advantage—to what is merely recondite. We wish this Cyclopædia every success, and shall take care to notice whatever may be novel or valuable in its pages.

The first part contains the articles—Abdomen—Abortion—Abscess—Acupuncture—Adhesion, Adhesive Plaster, and Aide—Albugo,—Alese,—Alum—Alveoles—Alvine Concretion—Amaurosis—and Ambulance—with some other minor articles which it is unnecessary to particularise.

Perhaps the best article is that on Amaurosis by Mr. Tyrell. To this we shall refer in another place.† The subject of Alvine Concretions is handled by Dr. Alexander Monro, in a not uninteresting manner, and we shall present an account of his paper.

*Alvine or Alimentary Concretions.* Dr. Monro excludes biliary concretions from consideration, unless they constitute the nuclei of the alvine of which he treats.

---

* We regret to say that we have not received many of the previous parts. We shall endeavour, as opportunities occur, to take a more particular notice of this work.

† Vide the notice of Middlemore on the Eye.

Substances, he says, of this description have been occasionally found within the stomach or intestines of man or quadrupeds after death; or they have been discharged during life by the effort of vomiting, or by straining at stool. Such substances are not equally common in every part of the alimentary tube; their most common seat is at the commencement of the larger intestine, near to its termination in the anus, or in the sigmoid flexure of the colon, where they have been frequently lodged for a very considerable time, obstructing, to a more or less extent, the passage of the fæcal matter, thereby deranging the functions of the digestive apparatus, and ultimately causing great distention of the bowels, which is often succeeded by acute pain, febrile symptoms, inflammation, ulceration, and the death of the animal.

Dr. Mason Good speaks in his Nosology of enterolithes and of stony concretions in the stomach or intestinal canal. He would seem to look on these as the results of morbid action, occasioning the deposition of phosphate of lime. Dr. Monro justly views this as a too exclusive notion, and informs us that he has not had an opportunity of examining any concretion in the alimentary canal answering precisely to Dr. Good's description of the enterolithes.

The inferior animals, from the nature of their food and of their tegumentary coverings, are more liable to alimentary concretions than man is. These are not unfrequent amongst horses, oxen, deer, and other quadrupeds that live on grass and other vegetable matter. That their production is connected with the covering of the animal is obvious from the fact that those concretions or balls, as they are commonly called, are by no means uncommonly met with in the stomachs of horses, oxen, calves, and deer, and these are found to be composed chiefly of hairs corresponding in all respects to those on the skin of the animal, and cemented together by the mucus and saliva of the throat.

It is reasonable to suppose that the kind of food must exert much influence on the formation of these extraneous bodies, and facts appear to bear out the conclusion. These concretions, for example, are more common in Scotland, where oaten bread is used, than in any other part of Europe. Dr. Johnson's definition of oats* is no doubt in the main a true one, and our Caledonian peasantry suffer from their coarse and homely diet. Dr. Monro has had abundant opportunities of examining the stools of his countrymen, and the result is such a classification of the various sorts of concretions found in them, as must excite the admiration of all other people.

"The concretions found in the alimentary canal vary considerably in their physical and chemical properties, and may be classed as follows: The first class comprehends such concretions as are formed of a number of very small vegetable fibres, being composed chiefly of the husk of the oat, held in union by a tenacious mucus; in some concretions of this description, the centre or nucleus is sometimes found to be a cherry or prune stone, a small fragment of wood or bone, or a little gravel; and, according to some authors, a small biliary concretion, or a small portion of adipocire. In some, but by no means in all such concretions, there are a few white, thin, concentric, laminæ, which are either phosphate of lime, or the ammoniaco-magnesian phosphate. When such concretions have attained a considerable size they are of irregular shape, sometimes

---

* Food for horses in England, and for men in Scotland.

tuberculated, and resembling a mulberry, and sometimes incrusted by a coating of phosphate of lime, or ammoniaco-magnesian phosphate, of two or three lines in thickness.

The second class of concretions, which is much more rare than the first, bears some resemblance to it, being composed of a number of cherry or prune stones or gooseberry seeds cemented together by a tenacious mucus. Concretions of this description have also been described by Mr. Children, (Phil. Trans. 1822,) and he has given a case where such a concretion proved fatal a very short time after the patient had eaten a large quantity of plums.

The third class differs essentially from the two preceding, being composed of vitiated bile, saliva, or pancreatic juice, and are not primarily formed within the alimentary canal, but find their way into it through the ducts proper to the salivary glands, pancreas, or liver.

The fourth class comprehends those concretions which are composed of a cetaceous substance: they have neither the specific levity, crystallized form, colour, nor bitter taste of gall stones, and are probably formed in the intestinal canal."

The fifth class comprehends those concretions, the nature of which was discovered by Dr. Kennedy of Glasgow, (see *Medico-Chir. Journal* for Sept. 1817),and which Dr. Ure has described as similar in colour to ambergris.

" The sixth class comprehends such concretions as are composed of magnesia or chalk, and which have been found in the alimentary canal of persons who for sometime have been in the habit of taking considerable quantities of these earths.

The seventh class is that which comprehends those concretions which are described as having been discharged by vomiting, by persons who had suffered severely from gout; but of these I know nothing, not having yet seen them.

The last class of concretions found in the human alimentary canal is almost entirely formed of the fibrine of the blood, which is incorporated with a certain proportion of the red globules, and occurs sometimes when the mucous membrane of the intestinal tube has been attacked by acute inflammation, as in severe cases of dysentery."

Dr. Monro having enumerated the preceding varieties of alimentary concretion, confines his descriptions chiefly to the first. His observations upon these are mainly founded on the large collection of them in the Anatomical Museum belonging to the University of Edinburgh. He examines them successively under the relations of number, size, figure, colour, structure, or chemical composition. He then investigates the causes which give rise to their formation, the symptoms which attend them, and the treatment medical and surgical adapted for them. We shall follow Dr. Monro through these sections of his inquiry—as, probably, the majority of medical men are very imperfectly acquainted with the subject.

A. *Number*.—One or two concretions only are usually found in the human alimentary canal. But Dr. Monro's father was accustomed, in his lectures, to make mention of the case of a boy of twelve years of age, in whose colon he could distinctly feel twelve concretions of different sizes, and these were moveable. Dr. Malcolm, of Perth, sent our author two, of sixty concretions which he took from the same individual, some of the smaller having been discharged by the anus, and the larger being found after the patient's death impacted firmly in the large intestines. Some other instances of numerous calculi are quoted. It is singular that, though these are so rife at Edinburgh, they are scarcely to be met in Glasgow. Professor Burns has not only never seen a case of this description, but he has never known any one who had seen one.

B. *Size*.—" The size of these concretions is extremely *various*. Some do not exceed that of a garden pea; but others are as large as an orange. My grandfather has described several he met with, as being five, six, seven, and even eight inches in circumference; and from the body of a woman, who consulted my father, a concretion was taken out of the colon, three years after by Mr. Renton, surgeon, in Pennycuick, which weighed four pounds." 69.

C. *Figure.*—The large concretions are more irregular in size than the small, and some seem to be made up of a congeries of others. Some alvine concretions are perforated by a number of small holes like certain corals. The smaller concretions are somewhat of an oval or spherical shape, slightly flattened at the sides, which is probably occasioned by the friction and pressure of one concretion upon another; and some impress each other so much as to resemble a cup and ball.

D. *Colour.*—The colour of the surface of the smaller calculi is an ochreish yellow—that of the larger coffee-like, or purple, or, in some instances, whitish.

E. *Structure.*—" The human alvine concretions which were collected by my father, are, with a few exceptions, similar as to structure; all are more or less porous, and somewhat like dried sponge; when examined by the aid of a good magnifying glass, they seem to be made up of a number of very small fibres, intimately interwoven with each other, like those in a hat, or in chamois leather, and the interstices between the fibres are filled up by earthy matter.

In consistence, these concretions resemble dried sponge, and like it, may be cut with a sharp knife; but when a blunt knife is used, the dried concretion crumbles to pieces. There are small holes on the surface of a few concretions, which pass for about half an inch into the substance of the calculus; and in one of the specimens there were twelve cavities of a cylindrical form, which penetrate as far as the very centre of the concretion, and were filled with a viscid mucous." 69.

The external crust of the larger concretions seldom exceeds two or three lines in thickness. Upon making a section of them, they are mostly found to be composed of distinct layers of a yellow or brown colour, but in some the layers do not pervade every part of the calculi; in some, there are no layers observed. But those found in the deer form an exception to the above observation; for some have a radiated structure, like biliary calculi. The lamellæ, which are about two lines in thickness, are generally of a lighter colour than the rest of the calculus, but sometimes they are darker. In the centre is usually some substance which has acted as a nucleus—a prune, or a cherry-stone, or a small piece of bone, or a biliary calculus. In some there are cavities, filled by small grains of sand adhering to each other by several small fibres. In the upper part of some of the concretions, many distinct white shining crystals are visible. By exposure to the air, the internal part of the concretion acquires a darker colour. Some further observations are made on the structure of these bodies, but we do not consider it necessary to pursue them.

F. *Chemical Analysis.*—No very unexceptionable analysis has hitherto been made of these concretions. To supply the deficiency, our author submitted several to Dr. T. Thomson for examination.

" 1st.—At first, they swim in water; but that is owing to their numerous pores being filled with air; as soon as it is expelled, they sink to the bottom, and remain there. The specific gravity I found to vary in different specimens from 1.376 to 1.540. I consider 1.400 as about the average specific gravity of the whole.

2nd.—When left in cold water, they soon communicate to it a brownish tinge. The water was found to have taken up the following substances :— 1. Albumen, which was separated in white flakes by boiling the water. 2. A brown substance, which I consider as peculiar ; it dissolved, at first, in water, but became nearly insoluble by the slow evaporation of the liquid ; it dissolved in alcohol ; it approached most nearly, in its properties, to vegetable extractive ; but the quantity which I obtained was too small for an exact examination. 3. Common salt, which crystallized when the water was allowed to evaporate spontaneously in an open vessel. 4. Phosphate of lime, which was precipitated by ammonia. 5. Sulphate of soda, in a very minute proportion. 6. Perhaps, also, sulphate of lime ; but the quantity of this salt must have been very small.

3rd.—Alcohol dissolved the peculiar brown matter, and some of the salts, but extracted nothing particular.

4th.—Potash ley separated the albumen, the brown matter, and perhaps some of the salts.

5th.—Muriatic acid separated a notable proportion of phosphate of lime.

6th.—After the action of all these re-agents, there remained behind a peculiar substance, having the colour and texture of the calculus. Ten grains of calculus left 1.2 grains of this matter ; it was very light, having the appearance of cork, or rather, of the peculiar fungus, which is used on the Continent for tinder, and which the French call *amadou ;* it was in very short threads. This substance is tasteless, insoluble in water, alcohol, æther, potash ley, and muriatic acid ; it blackens sulphuric acid, and is dissolved, being partly reduced to charcoal. In nitric acid it dissolves very slowly, and only when assisted by heat, and hardly effervesces. When the solution is evaporated to dryness, a whitish residue remains, which has a bitterish taste, and is imperfectly soluble in water. Nitric acid does not convert it into any of the vegetable acids, though digested on it repeatedly. This substance burns with a slight flame, and rather like a vegetable than an animal body. It is undoubtedly of a peculiar nature, differing from every animal and vegetable substance hitherto examined. Its insolubility in potash ley distinguishes it readily from wood. It has no resemblance to any animal substance whatever.

7th.—The calculi consist essentially of alternate layers of this peculiar substance, and of phosphate of lime. Sometimes the substances are intimately mixed, instead of being in alternate layers. The albumen and brown matter seem to serve as a cement ; the other substances are in a small proportion. The crust on the outside of some of the calculi consists of phosphate of lime, mixed with a brown animal matter. In a few specimens I observed crystals of phosphate of ammonia and magnesia, upon the outside crust of the calculi ; but these appearances are uncommon."

The calculi are clearly insoluble by any substances which could be applied to them in the living body. Though the composition of different specimens varies, they all contain that fibrous substance which Dr. Thomson considers a peculiar substance. Though analysis does not clear up all obscurity with respect to their formation, we may yet suppose that they are occasioned by the remains of ingesta, round which are deposited saline and other ingredients derived from the food and the secretions.

*Causes of Alimentary Concretions.*—To these we have already alluded. The indigestible debris of the aliment usually constitute the nucleus, while the irritated or the weak mucous membrane, by its vitiated secretions contributes to the increase of the calculus. Excessive doses of magnesia have given rise to concretions.

H. *Symptoms.*—" Alvine concretions prove a source of much irritation, and impair the functions of the stomach and intestines, exciting pain of variable intensity, sensibility of the entire abdomen, nausea, and severe vomiting ; distention of the alimentary tube, inflammation, looseness with much griping, or constipation with tenesmus and tormina, hiccup, &c., the symptoms having a general resemblance to those produced by a strangulated hernia. The pain in the bowels is, in some cases, fixed to one part, being much more severe at one time than another, especially after taking acids, or food of difficult digestion; the ingestion of these substances is frequently followed by nausea and vomiting; dry skin; furred tongue; quick pulse, and other febrile symptoms, which, however, are transitory. Some patients are constipated for two or three days, and have, notwithstanding, a constant inclination to go to stool. Such is the case, more particularly, when the foreign body is stopped in the colon, or rectum, the fixed position, and distensibility of which, favour its stoppage. In these cases, there is not only the impossibility of voiding the excrements, but sometimes the urine itself is retained. The belly is meteorized, the colic pains and vomiting are severe; sometimes there is hæmorrhage, or oozing of purulent matter from the anus; if no remission of the symptoms can be procured, enteritis, peritonitis, and gangrene, supervene. Others have watery stools, and discharge with them a quantity of viscid ropy mucus, or blood, after which they are much relieved. Some patients who for years have been afflicted by this disorder, suffer much from a burning sensation at the pit of the stomach, and some discharge their stools involuntarily.

Upon relaxing the parietes of the abdomen, a very hard, painful, globular tumour may generally be felt at the origin, or in the course of the large intestines; and very often at the sigmoid flexure of the colon.

The concretion is generally immovable within the intestine; but often appears to change its place in consequence of the change of place of the intestine which contains it. Hence the change appears greatest where the concretion is in the small intestines, or arch of the colon, which, from the length of mesentery, or meso-colon, are very moveable." 73.

Ultimately the patient is quite reduced and worn out. Concretions have, in some cases, remained for years before they have been dislodged. When they are very large, and lodged, either in the transverse arch, or sigmoid flexure of the colon, they may be distinctly perceived. When the concretion passes into the rectum, it creates excruciating pain in the region of the pelvis and fundament, especially when the patient goes to stool, and the extraneous body may then be perceived during the effort of straining at stool. In fortunate cases it is either ejected by vomiting, or discharged by stool, or extracted from the rectum.

Tumors occurring in the tract of the alimentary canal may be mistaken for alvine concretions. Amongst those tumors we may enumerate such as spring from the omentum or the mesentery, incipient dropsy of the ovaries, anormal position of one of the kidneys, and scirrhous pylorus. The diagnosis of these or of other tumors must hinge on precise investigation of their nature, a close inquiry into their history, and an accurate general acquaintance with pathology. It is idle to detail two or three cases, or to attempt by their means to elucidate diagnoses which can only be based on extensive knowledge. The only case we would extract is that which follows :—

*Case.* "I was desired by the late Dr. Kilgour of Musselburgh, to visit a man of seventy-three years of age, much emaciated, suffering very acute pain in the abdomen, which was much increased on pressure, and who had had no passage from his bowels for several days. His pulse was quick and hard. The relations informed us that he had been for many years subject to costiveness and stomach complaint. Upon examining the abdomen, several hard substances were distinctly perceived by the touch, a little above the left ilium; these hard substances, it was stated, had been distinctly felt by Mr. Cunningham, surgeon, at Tranent, and also by themselves, for eight or ten years.

Venesection, and large doses of purgatives were employed, but in vain; the patient died in six days after I visited him.

His body was opened by my assistant, Mr. Fyfe, in the presence of Dr. Kilgour and myself. All the viscera of the abdomen were found in a sound state, excepting the sigmoid flexure of the colon, on which there were several black spots; it contained a mass, which, on being minutely examined, proved to be fæces, very much indurated, and collected into a number of hard round tumours." 73.

Dr. Monro particularly dwells upon a point which he deems of much importance:—that alvine concretions may at first be moved from one part of the alimentary canal to another, but that at length they become immoveable. This circumstance he thinks both marks the progress of the malady, and determines the chance of dislodging the concretion.

If the concretion is not voided or removed, the obstruction of the intestines continues to augment, inflammation is set up, and the patient dies.

*Appearances on dissection.*—In the milder cases, the intestines are found to be considerably dilated, above the place where the concretion has been lodged, and in some there is a distinct sac, the coats of which attain a considerable thickness. In one instance, the ileo-cæcal valve had disappeared. The mucous membrane of the sac was much thickened and corrugated, so that the cavity which lodged the concretion seemed to be formed by a dilatation of the extremity of the ilium. The villi on the mucous membrane were not perceptible. Owing to the irritation of the concretion, the intestine is sometimes found contracted above and below the seat of the concretion; and in the cases described by Monro, Turner, and also by Horstius, the concretion adhered to the inner surface of the sac. Where death has been occasioned by inflammation, this may have extended to a greater or less distance, and produced its usual and familiar consequences. In a very few cases, ulceration of the intestinal canal has permitted the concretion to escape into an abscess.

*Treatment.*—Dr. Monro favours us with very circumstantial directions on the mode of treatment, when concretions have given birth to colic, constipation, inflammation, and so forth. But we need not detain our readers by repeating them. An acquaintance with the general principles of medicine would suggest them. Perseverance in oily and emollient purgatives, and glysters, is probably as little adapted to do harm, and as much adapted to do good, as any other method of management. Dr. Monro quotes from his father's memorandum a case in which this plan was pursued, and would perhaps have been in the end successful, if the patient had not died of the concretion in the interim.

*Treatment by an operation.*—When the concretion is low down in the rectum it may be removed by forceps introduced into the bowel through the anus. This has been several times done with success.

But when the concretion is not within reach of an instrument introduced through the anus, it has been proposed to cut into the bowel, and remove the calculus through the wound. Dr. Monro secundus recommended the performance of the operation, by a vertical incision, extending from the twelfth rib to the upper part of the ilium—an incision by means of which the colon might be opened behind the peritoneum, and without injury to it. Others have advocated making an artificial anus in the groin. And others, "going the whole hog," have advised the surgeon to cut into the belly direct, and not to care a rush for the peritoneum.

When we reflect that the existence of concretions is not generally indicated by conclusive symptoms—that when it is so, the concretion *may* not be in that portion of the colon which is accessible without a wound of the peritoneum—that the risk of any operation must be great, its performance difficult, and the issue dubious—when we reflect upon all these circumstances, we think we may predict that gastrotomy will seldom be performed for alimentary concretions, and if performed, that it will be so oftener than it will succeed.

